# Calcium-sensing receptor-mediated macrophage polarization improves myocardial remodeling in spontaneously hypertensive rats

**DOI:** 10.3389/ebm.2024.10112

**Published:** 2024-04-05

**Authors:** Jiaqi Zhao, Ning Lu, Yuanyuan Qu, Wei Liu, Hua Zhong, Na Tang, Jiayi Li, Lamei Wang, Dongmei Xi, Fang He

**Affiliations:** ^1^ Key Laboratory of Education Ministry of Xinjiang Endemic and Ethnic Diseases, NHC Key Laboratory for Prevention and Treatment of Central Asia High Incidence Diseases, Department of Pathophysiology, School of Medicine, Shihezi University, Shihezi, Xinjiang, China; ^2^ School of Medicine, Tarim University, Alaer, Xinjiang, China; ^3^ Department of Respiratory Medicine, The First Affiliated Hospital of Shihezi University School of Medicine, Shihezi, Xinjiang, China

**Keywords:** calcium-sensing receptor, essential hypertension, remodeling, macrophage polarization, NLRP3 inflammasome

## Abstract

Chronic inflammation is a key element in the progression of essential hypertension (EH). Calcium plays a key role in inflammation, so its receptor, the calcium-sensing receptor (CaSR), is an essential mediator of the inflammatory process. Compelling evidence suggests that CaSR mediates inflammation in tissues and immune cells, where it mediates their activity and chemotaxis. Macrophages (Mφs) play a major role in the inflammatory response process. This study provided convincing evidence that R568, a positive regulator of CaSR, was effective in lowering blood pressure in spontaneously hypertensive rats (SHRs), improving cardiac function by alleviating cardiac hypertrophy and fibrosis. R568 can increase the content of CaSR and M2 macrophages (M2Mφs, exert an anti-inflammatory effect) in myocardial tissue, reduce M1 macrophages (M1Mφs), which have a pro-inflammatory effect in this process. In contrast, NPS2143, a negative state regulator of CaSR, exerted the opposite effect in all of the above experiments. Following this study, R568 increased CaSR content in SHR myocardial tissue, lowered blood pressure, promoted macrophages to M2Mφs and improved myocardial fibrosis, but interestingly, both M1Mφs and M2Mφs were increased in the peritoneal cavity of SHRs, the number of M2Mφs remained lower than M1Mφs. *In vitro*, R568 increased CaSR content in RAW264.7 cells (a macrophage cell line), regulating intracellular Ca^2+^ ([Ca^2+^]_i_) inhibited NOD-like receptor family protein 3 (NLRP3) inflammasome activation and ultimately prevented its conversion to M1Mφs. The results showed that a decrease in CaSR in hypertensive rats causes further development of hypertension and cardiac damage. EH myocardial remodeling can be improved by CaSR overexpression by suppressing NLRP3 inflammasome activation and macrophage polarization toward M1Mφs and increasing M2Mφs.

## Impact statement

Essential hypertension is a multifactorial chronic cardiovascular disease that has been clinically addressed by pharmacological interventions targeting the renin-angiotensin system, but some patients still respond to this pharmacological treatment with resistance. This study provides convincing evidence that CaSR can attenuate hypertension-mediated myocardial remodeling. We further demonstrate that the beneficial function of CaSR was achieved by regulating macrophages/NLRP3 inflammasome. We therefore found a new mechanism of cardioprotective effect of CaSR, one which also offers a novel theoretical basis for the therapy of hypertension-induced myocardial remodeling.

## Introduction

Hypertension is believed to be a chronic low-grade inflammatory process with multifactorial effects [1]. The role of the immune response in the pathogenesis of hypertension is very important. The role of calcium-sensiing receptor (CaSR), among G protein-coupled receptors, in cardiovascular disease processes has been extensively investigated. As part of the innate immune system, CaSR is involved in immunomodulatory processes by binding to its metabolic regulators in response to tissue injury and inflammation. Decreased serum parathyroid hormone, 1,25-dihydroxy vitamin D, and calcium levels promote calcium homeostasis [2]. Whether it acts through immunomodulatory functions in myocardial tissue remains unknown.

In hypertensive patients, the normal structure of the vascular endothelium is disrupted by prolonged high pressure and abnormal blood flow, resulting in the attachment of inflammatory cells and the activation of the monocyte-macrophage system to produce inflammatory factors [3]. The functional classification of macrophages (Mφs) in the inflammatory state includes a binary classification of activated and alternatively activated Mφs, deriving these types of Mφs into M1 and M2 types under nonpathogen-driven conditions [4]. During homeostasis, both M1 macrophages (M1Mφs) and M2 macrophages (M2Mφs) are present in the vasculature and heart. Intravital microscopy shows M1Mφs circulate rapidly whereas M2Mφs circulate more slowly, crawling along the endothelium [5]. The cardiac Mφs play a role in cardiac development, immuno-surveillance and may contribute important specialised cardiac functions, such as conduction, though their exact functional significance is still emerging [6]. Following acute myocardial infarction, macrophage populations expand at the site of infarction and change their phenotype dramatically in the murine heart [5].

Activated inflammasomes have the ability self-differentiation, producing active forms of caspase-1, cleaving pro-interleukin 1β (IL-1β) and pro-interleukin 18 (IL-18), and releasing active IL-1β and IL-18 [7]. NOD-like receptor family protein 3 (NLRP3) inflammasome is also considered as the most important isoform causing inflammation in chronic diseases. Dalekos [8] examined several hypertensive patients and observed an overall increase in serum levels of IL-1β, which is activated by the upregulation of type 1 angiotensin receptor expression, thereby enhancing and involving in the process of hypertension [9]. In another experiment, it was shown that an increase in extracellular calcium can bind through CaSR, activate CaSR, initiate relevant ion channels, and allow calcium (Ca^2+^) entry into the cell, which in turn causes Ca^2+^ release in the endoplasmic reticulum, stimulates inflammasome assembly, activates the effector protein caspase-1, and releases the proinflammatory cytokine IL-1β after maturation [10]. However, the role of CaSR-mediated NLRP3 inflammasome activation in hypertension has not yet been investigated.

In this study, CaSR activity was enhanced or attenuated by R568 (a CaSR agonist) and NPS2143 (a CaSR antagonist), while NLRP3 inflammasome activity was inhibited by MCC950 (an NLRP3 antagonist). The effects of CaSR on blood pressure and myocardial remodeling in spontaneously hypertensive rats (SHRs) were investigated at the overall tissue and cellular levels. In addition, its association with NLRP3 inflammasome and macrophage polarization was discovered.

## Materials and methods

### Animals and treatments

SHRs and Wistar-Kyoto rats (WKY) were purchased from Beijing Viton Lihua Laboratory Animal Technology Co., Ltd. (Beijing, China). Male rats of 16 weeks, age-matched, and weighing approximately 290 g–310 g, were kept in an alternating 12 h light/dark cycle at a temperature of 25°C and constant humidity with free access to food and water. SHRs and WKY were randomly divided into the WKY group, SHR+normal saline (NS) group, SHR+R568 group, SHR+NPS2143 group, and SHR+R568+MCC950 group, with 10 rats in each group. Subsequently, daily intraperitoneal injections of R568 at 1.2 mg/kg/day [per 1 mg with 29 μL dimethyl sulfoxide (DMSO)] were administered [11]. NPS2143 was administered at 4.5 mg/kg/day (per 1 mg with 22 μL DMSO) [12], and MCC950 at 10 mg/kg/day [13]. All compounds were purchased from Tocris Bioscience R&D Systems (Minneapolis, MN, United States). All animal procedures were conducted with the approval of the Animal Care and Use Committee of Shihezi University (Shihezi, China; approval number: A2020-164-01). Every effort was made to alleviate the animal’s suffering.

### Cell culture and treatment

The mouse macrophage cell line RAW264.7 was obtained from Peking Union Cell Center, Chinese Academy of Medical Sciences (Beijing, China) and was cultured in high sugar Dulbecco’s modified Eagle medium (DMEM, Gibco; United States) containing 10% fetal bovine serum (FBS, Gibco; United States). A penicillin-streptomycin suspension (Solarbio; Beijing, China) was added to prevent bacterial contamination; the cells were cultured at 37°C, 5% CO_2,_ and 100% humidity and carefully passaged before they reached confluence. Three to five generations of cells were collected for the experiment and divided into five groups: CON group; R568 group; NPS2143 group; R568+MCC950 group. R568, NPS2143 (5 μmol/L, per 1 μmol/L with 0.01 μL DMSO), and MCC950 (1 μmol/L) [14].

### Intraperitoneal mononuclear macrophage collection

Under chloral hydrate anesthesia, mononuclear macrophages were isolated from the peritoneal cavity of rats by injecting precooled phosphate buffered saline (PBS, Nakasugi Jinqiao; Beijing, China) into the peritoneal cavity. The cells were isolated by centrifugation at 1,000 rpm for 5 min. Then, the cells were cultured in DMEM containing 10% FBS at 37°C and 5% CO_2_ and allowed to differentiate.

### Quantitative real-time polymerase chain reaction (qRT-PCR)

Total RNA was extracted from each group of tissues, and 3 μg of total RNA was reverse transcribed to cDNA according to the manufacturer’s instructions (Tiangen Biotech; Shanghai, China). Data were analyzed using ABI7500 software (Applied Biosystems; CA, United States). PCR amplification (triplicates) was performed in a 20 μL reaction volume using SYBR Green/Fluorescein qPCR Master Mix (Thermo; United States). The reaction mixture without template cDNA was used as a negative control. The mRNA expression was normalized to the expression values of glyceraldehyde 3-phosphate dehydrogenase (GAPDH, endogenous control). The comparative CT method (ΔΔCT) determined the gene expression level. The primers for atrial natriuretic peptide (*ANP*), brain natriuretic peptide (*BNP*), β-myosin heavy chain (*β-MHC*), *CaSR*, *CD86*, *CD206* and *GAPDH* were as follows:

*ANP*: forward, 5′-CCT​GGA​CTG​GGG​AAG​TCA​AC-3′; reverse, 5′-ATC​TAT​CGG​AGG​GGT​CCC​AG-3′.
*BNP*: forward, 5′-TCC​TTA​ATC​TGT​CGC​CGC​TG-3’; reverse, 5′-AGC​CCA​GGA​TGC​CCT​TTA​GT-3′.
*β-MHC*: forward, 5′-GGC​CCT​TTG​ACC​TCA​AGA​AAG-3′;
reverse, 5′-GCC​ATT​CTC​TGT​CTC​AGC​GG-3′.
*CaSR*: forward, 5′-ACG​AGC​CTC​AGA​AGA​ATG​CC-3′; reverse, 5′-TCC​GCA​TCT​GCA​CAC​TGT​AG-3′.
*CD86*: forward, 5′-TTT​CGC​AGC​CCC​AGT​TTG​AT-3′; reverse, 5′-AAC​ACC​ACT​GTC​CTG​CTT​GG-3′.
*CD206*: forward, 5′-CTC​TAA​GCG​CCA​TCT​CCG​TT-3′; reverse, 5′-CAT​GAT​CTG​CGA​CTC​CGA​CA-3′.
*GAPDH*: forward, 5′-GAC​ATG​CCG​CCT​GGA​GAA​AC-3′; reverse, 5′-AGC​CCA​GGA​TGC​CCT​TTA​GT-3′.

### Flow cytometry analysis

Cells collected from the peritoneal cavity were observed under the microscope, nonadherent cells and blood cells were washed with PBS, cell scrapers were used to scrape the adhered cells (macrophages), and cells were collected by centrifugation at 1,000 r/min for 5 min. The cells were fixed with 4% paraformaldehyde at room temperature (RT) for 20 min and then with 3% bovine serum albumin (BSA) at 37°C for 30 min. The cells were stained with polarization markers [M1Mφ: anti-rat CD86-PE (Solarbio; Beijing, China); M2Mφ: anti-rabbit CD206-FITC (Solarbio; Beijing, China)], incubated at 37°C for 45 min, protected from light, and washed twice with PBS. The supernatant was discarded, 500 μL of PBS was added, and the samples were either stored at 4°C or directly analyzed by flow cytometry (BD Biosciences; CA, United States). The data were analyzed with FlowJo 10.4 software (BD Biosciences; CA, United States).

### Cell counting kit-8 assay (CCK-8 assay)

To analyze R568-induced cell viability, 5 × 10^3^ cells/well were seeded in 96-well microtiter plates and cultured in DMEM. Six wells per plate containing medium only served as blanks, and three wells containing untreated cells served as controls. Plates were incubated under standard cell culture conditions. RAW264.7 cells were treated with increasing concentrations (0–20 μmol/L, per 1 μmol/L with 0.01 μL DMSO) of R568. Cell viability was determined after different treatment times (1 h, 2 h, 4 h, and 6 h) using a CCK-8 (Apexbio; Houston, United States). The absorbance at 490 nm was measured using a microplate reader of model 680 (Bio-Rad Laboratories, Inc.; United States). The cell viability ratio was calculated using the following formula: Ratio = (A490 value in test group-A490 value in the blank group)/(A490 value in control group-A490 value in the blank group) × 100%.

### Flow cytometry analysis of [Ca^2+^]_i_ concentration

For the intracellular Ca^2+^ ([Ca^2+^]_i_) concentration, collecting by scraping with the cells (at 1 × 10^6^ cells/mL density) with a cell scraper, the cells were resuspended with the culture medium. A total of 1 µL Fluo-3 AM (5 μmol/L, Apexbio; Houston, United States) was added to each tube of the sample to be tested. Subsequently, the tube was mixed to allow full contact between the cells and the Ca^2+^ fluorescence probe, and then the cells were incubated at 37°C for 45 min. The cells were washed 2–3 times with Hank’s Balanced Salt Solution (HBSS, Gibco; United States) and the cells were resuspended by adding 500 µL HBSS. The Ca^2+^ concentration was detected with flow cytometry. The data were analyzed with FlowJo 10.4 software.

### Western blotting

Cells were washed three times with PBS and then lysed in lysis buffer (PMSF: RIPA, 1:100, Sigma-Aldrich; Merck KGaA, Germany). Tissues were excised and then lysed via sonication in a lysis buffer. After insoluble debris was pelleted by centrifugation at 12,000 ×g for 15 min at 4°C, the supernatants were collected, and protein concentrations were assessed by the bicinchoninic acid method. The samples were fractionated on 10% sodium dodecyl sulfate-polyacrylamide gel electrophoresis (SDS-PAGE) gels, transferred to polyvinylidene fluoride membranes (EMD Millipore; Merck KGaA, Germany), and blocked with 5% non-fat milk or BSA for 2 h at RT.

### Immunofluorescence analysis

Cells: Cells were seeded on sterile coverslips kept in 24-well multiwell plates. The following day, the cells were washed with PBS and fixed in 4% formaldehyde for 20 min. Cells were washed with PBS three times before incubating the cells with 3% BSA for 30 min at 37°C to minimize nonspecific binding. Cells were incubated with CD86 and CD206 primary antibodies (1:100 in the 3% BSA blocking buffer, Nakasugi Jinqiao; Beijing, China) overnight at 4°C. The following day, the samples were washed and exposed to fluorescein isothiocyanate (FITC, Solarbio; Beijing, China) and tetramethylrhodamine isothiocyanate (TRITC, Solarbio; Beijing, China) fluorochrome-conjugated secondary antibodies (1:50) in the dark for 1 h. The cells were then incubated with 4′,6-diamidino-2-phenylindole (DAPI) at 37°C for 20 min. Finally, the cells were observed using confocal microscopy, and images were captured.

Tissues: The dewaxed, rehydrated tissue sections were transferred into antigen retrieval buffer solutions and placed in a microwave, blocking solution was added for incubation, and the immunostaining procedure was initiated immediately. The CD86-specific primary antibodies were diluted in PBS and incubated at 4°C overnight. The horseradish peroxidase (HRP)-labeled secondary antibody was incubated for 50 min at RT. After two washes with tris buffered saline containing Tween 20 (TBST), Cy5 fluorescent antibodies were incubated in the dark for 10 min at RT. The samples were then heated in the microwave, and the primary anti-CaSR antibody (Abcam; United States) was diluted and incubated overnight at 4°C. HRP-labelled secondary antibodies were incubated at RT for 50 min, washed with TBST, and incubated in the dark with FITC fluorescent antibodies at RT for 10 min. After heating, anti-CD206 was incubated at 4°C overnight. HRP-labelled secondary antibodies were incubated at RT for 50 min and washed with TBST. Cy5 fluorescent antibodies were incubated at RT for 10 min in the dark. Subsequently, the slides were mounted with DAPI for nuclear staining. Finally, the slides were incubated at RT for 15 min with an autofluorescence quencher to quench the tissue autofluorescence. Slides were stored at 4°C and scanned the following day (DAPI excitation wavelength: 330–380 nm, emission wavelength: 420 nm, blue light; FITC excitation wavelength: 465–495 nm, emission wavelength: 515–555 nm, green light; Cy3 excitation wavelength: 510–560 nm, emission wavelength: 590nm, red light; Cy5 excitation wavelength: 608–648 nm, emission wavelength: 672–712nm, pink light).

The following methods [measurement of blood pressure (BP), assessment of cardiac function, tissue collection and measurement of the heart-to-body weight ratio, hematoxylin and eosin staining (H&E), masson staining] are referenced but not limited to *DOI: 10.1177/1535370219854325.*

### Statistical analyses

The results are expressed as the mean ± SE.s. Using SPSS 26.0 software (IBM Corp., United Sates), experimental groups were compared using one-way ANOVA, followed by Bonferroni correction. The statistical significance was indicated by *p* < 0.05.

## Results

### R568 reduces BP, and NPS2143 increases BP in SHRs

**FIGURE 1:**
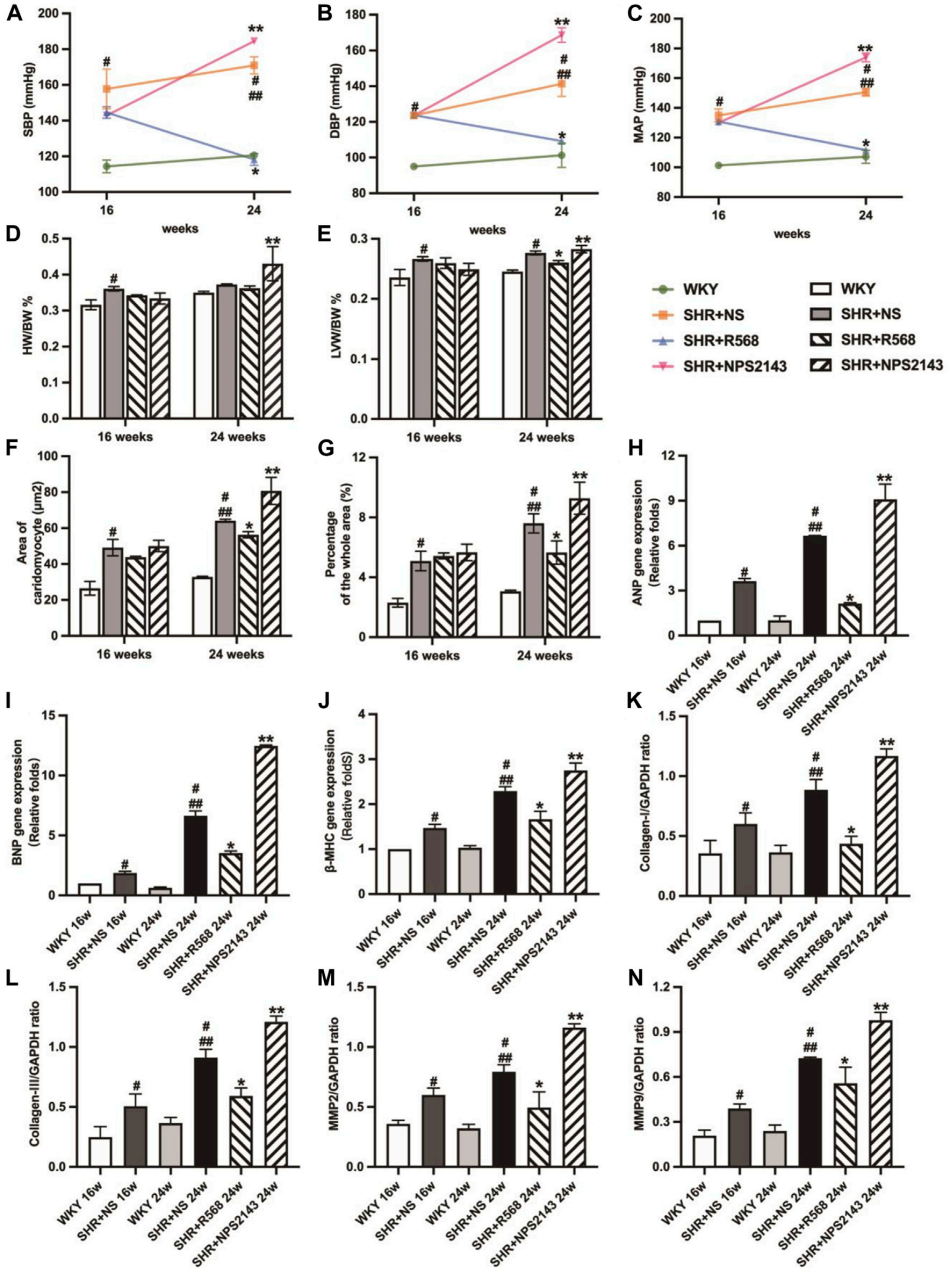
Comparison of blood pressure in each group evaluation of myocardial hypertrophy and fibrosis. **(A)**: SBP; **(B)**: DBP; **(C)**: MAP; **(D)**: HW/BW; **(E)**: LVW/BW; **(F)**: quantitative analysis of the cell size (mm^2^) of cardiac myocytes; **(G)**: quantitative analysis of fibrosis; **(H–J)**: qRT–PCR of cardiac-specific fetal genes ANP, BNP and β-MHC; **(K–N)**: densitometric analysis of cardiac-protein expression. Values mean ± SE. s; *n* = 5; ^#^ *p <* 0.05, SHR+NS groups *vs.* age-matchedWKY groups; ^##^ *p <* 0.05, SHR+NS 24w group *vs.* SHR+NS 16w group; *p < 0.05, SHR+R568 groups *vs.* SHR+NS groups; **p < 0.05, SHR+NPS2143 groups *vs.* SHR+NS groups. HW/BW, heart-to-body weight ratio; LVW/BW, left ventricle-to-body weight ratio.

The average systolic BP (SBP), diastolic BP (DBP), and mean arterial pressure (MAP) were elevated in the SHR+NS groups compared to the age-matched WKY groups (*p* < 0.05). At 24 weeks, BP was higher than at 16 weeks (*p* < 0.05). However, BP was significantly reduced under R568 and increased under NPS2143 treatment at 24 weeks (*p* < 0.05; Figures 1A–C).

### R568 relieves cardiac hypertrophy and fibrosis, and NPS2143 exacerbates cardiac hypertrophy and fibrosis

In the SHR+NS groups, the difference in weight-to-body weight ratio (HW/BW) between SHRs and WKY rats disappeared with age (*p* > 0.05; Figure 1D), and left ventricular weight-to-body weight ratio (LVW/BW) increased significantly compared to the age-matched WKY rats (*p* < 0.05; Figure 1E). In addition, the R568 group exhibited a decreasing trend, while the NPS2143 group exhibited an increasing trend to the ratio compared with the SHR+NS group at 24 weeks (*p* < 0.05; Figure 1E).

The cross-sectional area of cardiocytes measured by H&E staining was greater in SHR+NS groups than in the age-matched WKY groups (*p* < 0.05), and the increasing area was correlated with age (*p* < 0.05). In addition, treatment with R568 reduced the area (*p* < 0.05), whereas treatment with NPS2143 increased the area after 8 weeks (*p <* 0.05; Figure 1F, Supplementary Figure S1A). Masson staining of heart tissue sections shows the intensity of collagen accumulation, as reflected by blue staining. Interstitial fibrosis was significantly increased in SHRs than in age-matched WKY rats (*p <* 0.05), and the area at 24 weeks was larger than that at 16 weeks (*p <* 0.05). However, SHRs treated with R568 exhibited decreased fibrosis (*p <* 0.05), whereas those treated with NPS2143 showed an increase in fibrosis (*p <* 0.05; Figure 1G, Supplementary Figure S1B).

Heart tissues of SHRs exhibited increased mRNA expression of ANP, BNP and β-MHC and protein expression of myocardial hypertrophy markers, including Collagen Ⅰ, Collagen Ⅲ, matrix metalloproteinase 2 (MMP 2) and matrix metalloproteinase 9 (MMP 9), compared with the age-matched WKY rats (*p <* 0.05). The expression of these proteins was upregulated in the heart tissues of 24-week-old rats compared to 16-week-old rats (*p <* 0.05). In contrast, SHRs treated with R568 exhibited significantly attenuated mRNA and protein levels (*p <* 0.05), and the treatment with NPS2143 markedly increased the mRNA and protein expression levels at 24 weeks (*p <* 0.05; Figures 1H–N, Supplementary Figure S1C).

### R568 improves LV functional parameters, and NPS2143 worsens LV functional parameters in SHRs

**FIGURE 2:**
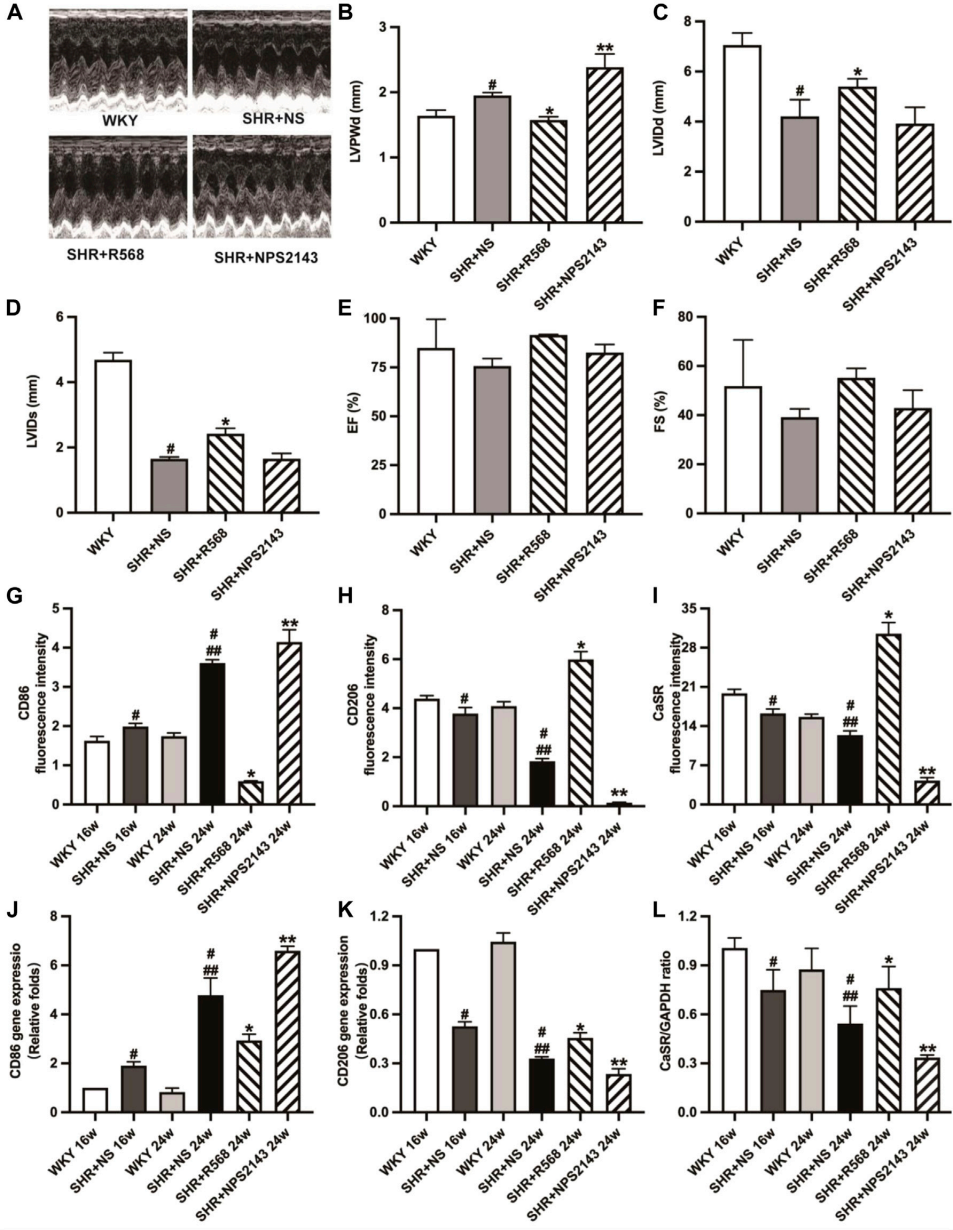
Detection of echocardiographic data and detection of macrophage surface markers and CaSR expression in myocardial tissue of each group of rats. **(A)**: images of M-mode of LV; **(B–F)**: LVPWd, LVIDs, LVIDd, EF, FS; **(G–I)**: quantitative analysis of immunofluorescence staining; **(J,K)**: qRT-PCR of macrophage surface markers (CD86, CD206) in myocardial tissues; **(L)**: quantitative analysis of western blotting (CaSR). Values mean ± SE. s; *n* = 5; ^#^ *p <* 0.05, SHR+NS groups *vs.* age-matched WKY groups; ^##^ *p <* 0.05, SHR+NS 24w group *vs.* SHR+NS 16w group; *p < 0.05, SHR+R568 groups *vs.* SHR+NS groups; **p < 0.05, SHR+NPS2143 groups *vs.* SHR+NS groups. LVPWd, left ventricular end-diastolic posterior wall dimension; LVIDs, left ventricular end-systolic diameter; LVIDd, left ventricular end-diastolic diameter; EF, ejection fraction; FS, fractional shortening.

Echocardiographic analysis showed significantly decreased left ventricular internal diameter systolic (LVIDs) and left ventricular internal diameter diastolic (LVIDd) and increased left ventricular posterior diameter (LVPWD) in SHR compared to age-matched WKY groups (*p <* 0.05). In addition, the R568 treatment reversed the changes in LVIDs, LVIDd, and LVPWd (*p <* 0.05). Treatment with NPS2143 enhanced the change in LVPWd, whereas there were no differences in LVIDs and LVIDd (*p* > 0.05). However, there was no difference in ejection fraction (EF) and fractioning shortening (FS) in any group (*p* > 0.05; Figures 2A–F).

### R568 increased the expression of CaSR and M2Mφs and decreased that of M1Mφs, while NPS2143 exerted the opposite effect in the cardiac tissue of SHRs

The immunofluorescence intensity of CD86, CD206 and CaSR in cardiac tissues analyzed by fluorescence microscopy displayed that SHRs had a higher expression of CD86 and lower expression of CD206 and CaSR (*p <* 0.05). The results were reversed after the R568 treatment (*p <* 0.05) and deteriorated after the NPS2143 treatment (*p <* 0.05; Figures 2G–I, Supplementary Figure S2A). Similar outcomes were observed in qRT-PCR (*p <* 0.05; Figures 2J, K) and western blotting analysis (*p <* 0.05; Figure 2L, Supplementary Figure S2B).

### The numbers of M1Mφs and M2Mφs were increased in the SHR peritoneal cavity, but the number of M2Mφs was lower than that of M1Mφs

**FIGURE 3:**
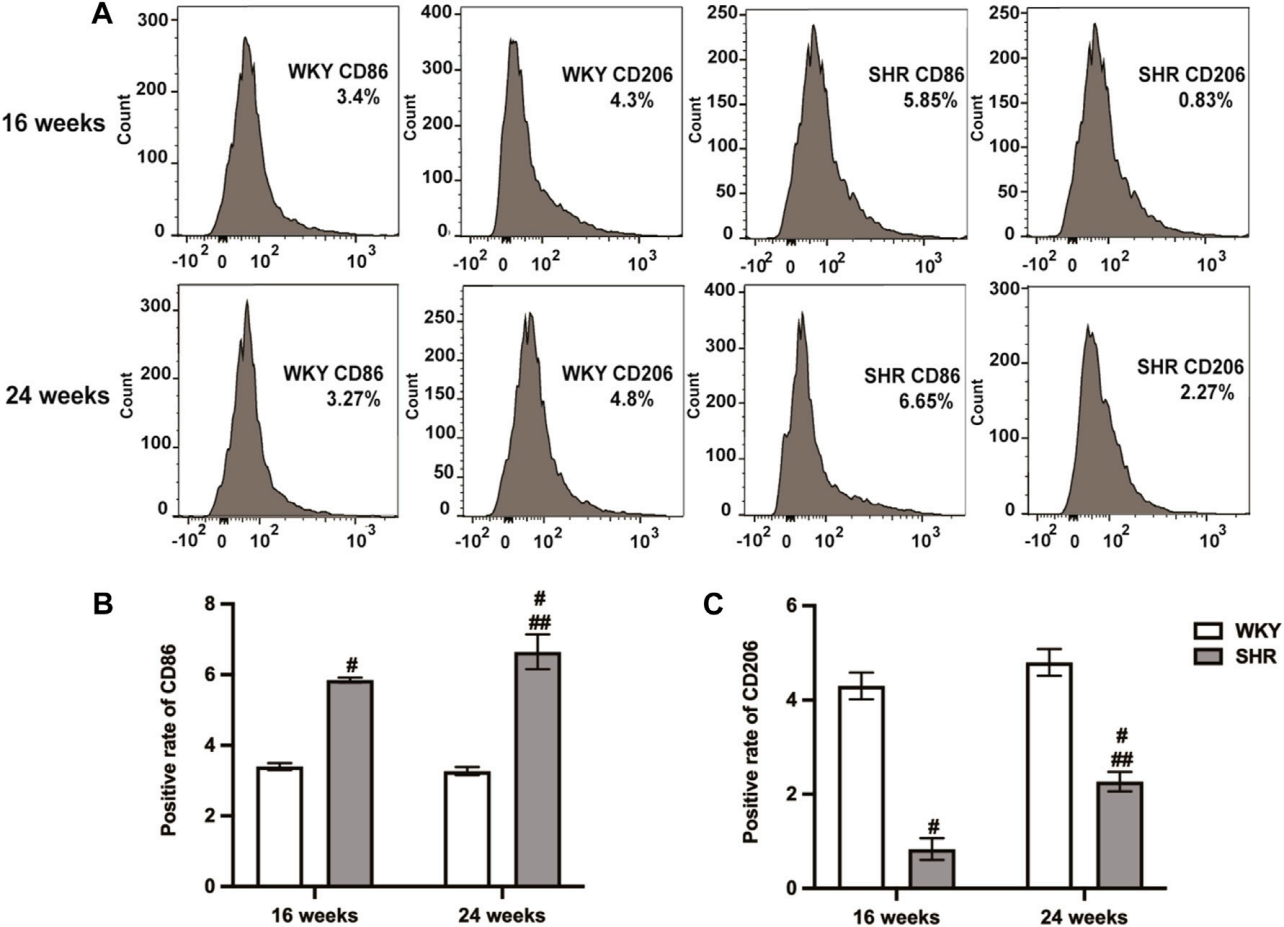
Types of peritoneal macrophages in each group of rats were detected by flow cytometry. **(A)**: peritoneal macrophages of 16-week-old and 24-week-old rats; **(B,C)**: quantitative analysis of **(A)**. Values mean ± SE. s; *n* = 5; ^#^ *p <* 0.05, SHR+NS groups *vs.* age-matchedWKY groups; ^##^ *p <* 0.05, SHR+NS 24w group *vs.* SHR+NS 16w group.

Flow cytometry analysis showed that CD86 was upregulated and CD206 was downregulated in SHRs compared to the age-matched WKY rats (*p <* 0.05). After 8 weeks, both CD86 and CD206 were upregulated (*p <* 0.05), whereas CD86 remained higher than CD206 (Figure 3).

### R568 increases CaSR expression, and NPS2143 decreases CaSR expression in RAW264.7 cells

**FIGURE 4:**
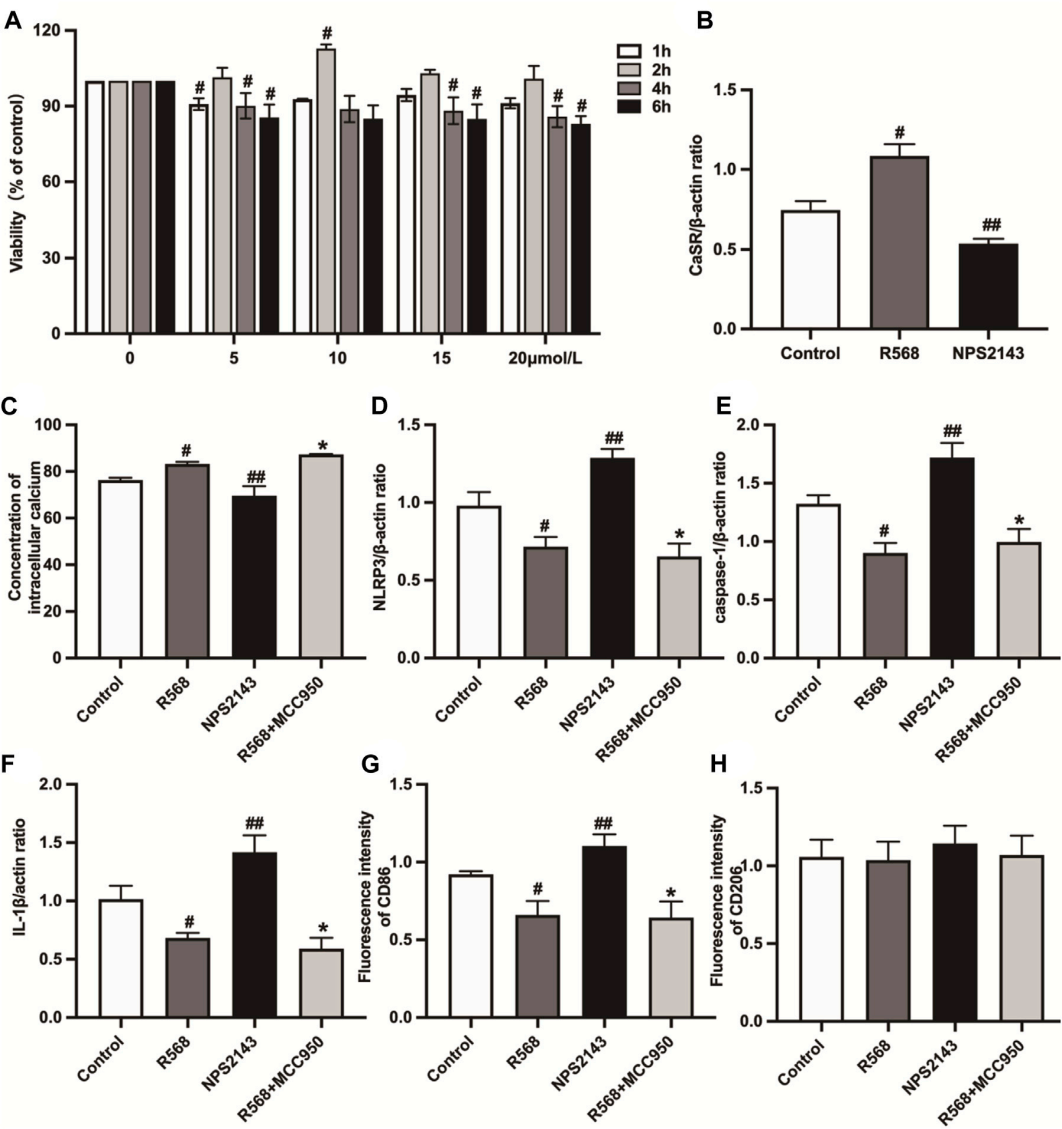
Detection of cell viability and the expression of CaSR, intracellular calcium concentration, NLRP3 inflammasome-related protein expression and macrophage types in RAW264.7 cells. **(A)**: Effects of R568 at different concentrations (5, 10, 15 and 20 μmol/L) for 1, 2, 4 and 6 h on the cell viability ratio of RAW264.7 cells as tested by CCK-8 assay; **(B)**: CaSR protein expression levels were assessed by western blotting analysis; **(C)**: quantitative analysis of [Ca^2+^]_i_; **(D–F)**: quantitative analysis of NLRP3 inflammasome-related protein (NLRP3, caspase-1, IL-1β); **(G**, **H)**: macrophage types by immunofluorescence staining. Values mean ± SE. s; *n* = 3; ^#^ *p <* 0.05, R568 group *vs.* Control group; ^##^ *p <* 0.05, NPS2143 group *vs.* Control group; *p < 0.05, R568+MCC950 group *vs.* Control group.

We determined the effect of different R568 concentrations and treatment durations on the cell viability of RAW264.7 cells. The results showed that RAW264.7 viability was enhanced by R568 treatment at 2 h, particularly at a 10 μmol/L concentration (*p <* 0.05; Figure 4A, Supplementary Figure S3A). Western blotting showed that compared to the control group, R568-treated RAW264.7 cells exhibited an abnormal upregulation of CaSR expression (*p <* 0.05), NPS2143-treated cells exhibited a significant downregulation (*p <* 0.05; Figure 4B, Supplementary Figure S3B).

### R568 inhibits NLRP3 inflammasome activation by regulating [Ca^2+^]_i_

Based on the relationship between [Ca^2+^]_i_ and NLRP3 inflammasome, the experiments measured the [Ca^2+^]_i_ concentration by Flou-3/AM and the activation of NLRP3 inflammasome in cells by western blotting under different drug treatments. The exposure of RAW264.7 cells to NPS2143 decreased [Ca^2+^]_i_ significantly compared with the control group (*p <* 0.05). However, treatment with R568 alone or the R568+MCC950 combination induced a significant increase in [Ca^2+^]_i_ compared to the control group (*p <* 0.05), but the difference was not statistically significant (*p* > 0.05; Figure 4C, Supplementary Figure S3D). Under NPS2143 treatment, the expressions levels of NLRP3 inflammasome-related molecular protein, NLRP3, caspase-1 and IL-1β were all upregulated (*p <* 0.05); under the treatment of R568 alone, R568+MCC950 combination, the protein expression levels were all downregulated (*p <* 0.05), and there was no significant difference between the two groups (*p* > 0.05; Figures 4D–F, Supplementary Figure S3C).

### R568 inhibits M1Mφs via NLRP3 inflammasome but has no effect on M2Mφs in RAW264.7 cells

Immunofluorescent staining was used to study some cell markers to investigate the influence of the CaSR-NLRP3 inflammasome on RAW264.7 cells. RAW264.7 cells were fluorescently stained and evaluated for CD86-and CD206-positive signals after different treatments. Treatment with NPS2143 enhanced staining for CD86, whereas decreased staining intensity between R568 alone and R568 combined with MCC950 (*p <* 0.05) did not differ between the two groups (*p* > 0.05). However, all groups had no significant difference in CD206 (*p* > 0.05; Figures 4G, H, Supplementary Figure S3E).

### R568 improves cardiac function and myocardial fibrosis via NLRP3 inflammasome in SHRs

**FIGURE 5:**
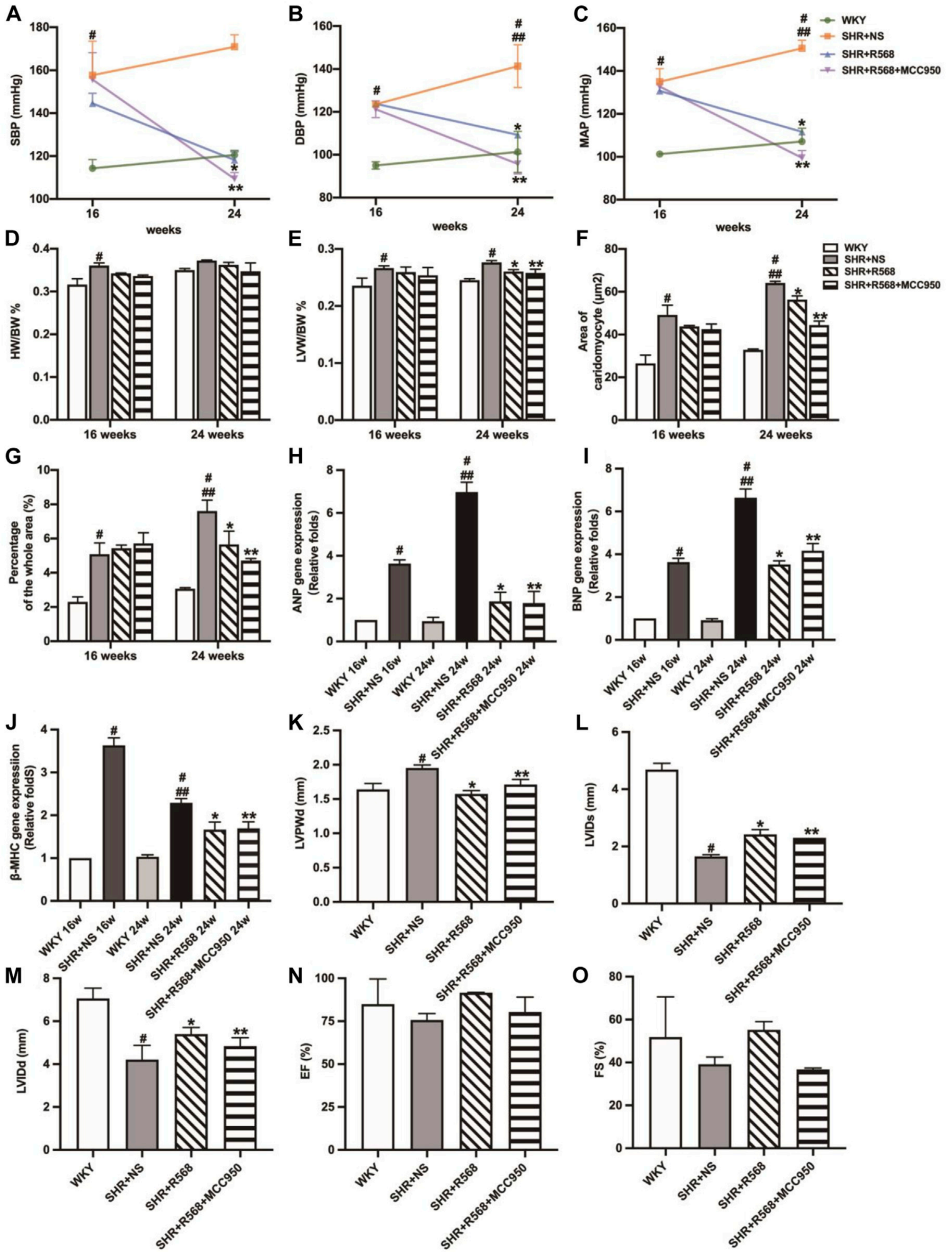
Evaluation of BP, myocardial hypertrophy and fibrosis between different groups. **(A)**: SBP; **(B)**: DBP; **(C)**: MAP; **(D)**: HW/BW; **(E)**: LVW/BW; **(F)**: quantitative analysis of the cell size (mm^2^) of cardiac myocytes; **(G)**: quantitative analysis of fibrosis; **(H–J)**: qRT-PCR of cardiac-specific fetal genes ANP, BNP and β-MHC; **(K–O)**: LVPWd, LVIDs, LVIDd, EF, FS. Values mean ± SE. s; *n* = 5; ^#^ *p <* 0.05, SHR+NS groups *vs.* age-matched WKY groups; ^##^ *p <* 0.05, SHR+NS 24w group *vs.* SHR+NS 16w group; *p < 0.05, SHR+R568 groups *vs.* SHR+NS groups; **p < 0.05, SHR+R568+MCC950 groups *vs.* SHR+NS groups.

In SHRs given R568 alone and R568 combined with MCC950 treatment for 8 weeks, there was a significant reduction in BP (SBP, DBP, MAP) in both groups (*p <* 0.05; Figures 5A–C), and LVW/BW was also significantly decreased (*p <* 0.05; Figure 5E). However, HW/BW remained unchanged (*p* > 0.05; Figure 5D). A significant reduction in cardiomyocyte cross-sectional area (*p <* 0.05; Figure 5F, Supplementary Figure S4A), collagen deposition area (*p <* 0.05; Figure 5G, Supplementary Figure S4B), and myocardial hypertrophy-associated mRNA expression (*p <* 0.05; Figures 5H–J) was observed in both groups. In addition, echocardiographic findings suggested a reduction in LVPWd and an expansion of LVIDs and LVIDd (*p <* 0.05; Figures 5K–M, Supplementary Figure S4C), whereas EF and FS remained significantly unchanged (*p* > 0.05; Figures 5N, O, Supplementary Figure S4C). All these changes were not significantly different in either of the drug-treated groups (*p* > 0.05).

### R568 inhibits M1Mφs and promotes M2Mφs via NLRP3 inflammasome in SHRs

**FIGURE 6:**
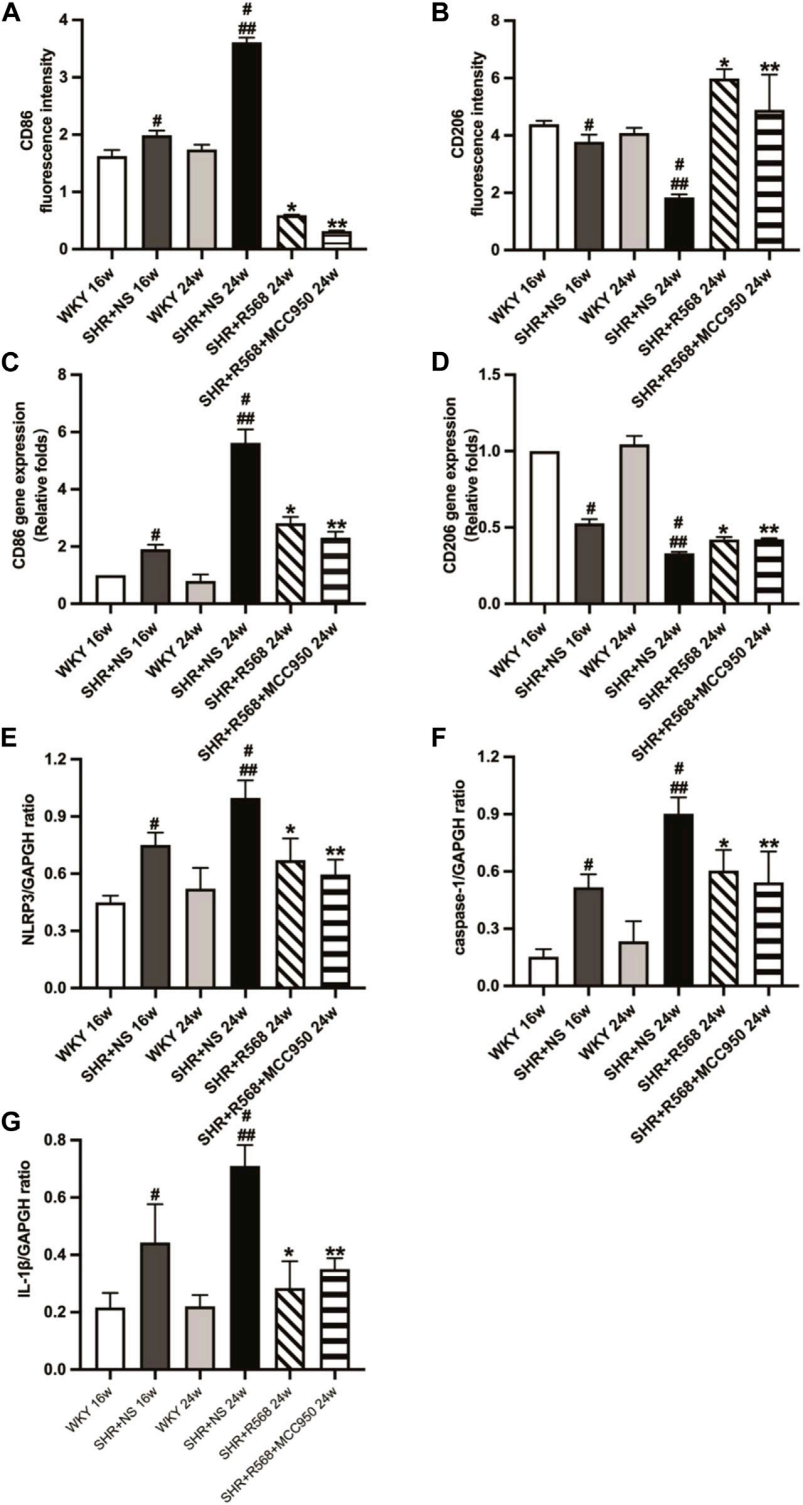
Detection of macrophage types and NLRP3 inflammasome-related protein expression of rats. **(A,B)**: macrophage types by immunofluorescence staining; **(C,D)**: Macrophage types by qRT-PCR; **(E–G)**: quantitative analysis of NLRP3 inflammasome-related protein (NLRP3, caspase-1, IL-1β). Values mean ± SE. s; *n* = 5; ^#^ *p <* 0.05, SHR+NS groups *vs.* age-matched WKY groups; ^##^ *p <* 0.05, SHR+NS 24w group *vs.* SHR+NS 16w group; *p < 0.05, SHR+R568 groups *vs.* SHR+NS groups; **p < 0.05, SHR+R568+MCC950 groups *vs.* SHR+NS groups.

During the study of macrophage polarization types in myocardial tissue, it was observed that the area of CD86 (M1Mφ) fluorescence in myocardial tissue was decreased, and the area of CD206 (M2Mφ) fluorescence was increased between WKY and age-matched SHR+NS groups (*p <* 0.05; Figures 6A, B, Supplementary Figure S5A). Similar results were observed in qRT-PCR (*p <* 0.05; Figures 6C, D). In addition, NLRP3 inflammasome activation was inhibited (protein expression levels of NLRP3, IL-1β and caspase-1 were reduced) after 8 weeks of R568 alone and in combination with MCC950 (*p <* 0.05; Figures 6E–G, Supplementary Figure S5B).

## Discussion

SHRs are frequently used in studies of essential hypertension, and disorders of the innate immune system can promote the development of high blood pressure in SHRs [15]. CaSR, as an extracellular Ca^2+^ receptor, is associated with initiating and progressing inflammatory responses. In addition, macrophage polarization and the NLRP3 inflammasome are associated with hypertension [16]. Our study demonstrates for the first time the protective effects of CaSR on EH-induced cardiac injury, activation of the NLRP3 inflammasome and changes in the type of polarization in macrophages.

CaSR activity can be modulated by ligands and chemoregulators involved in inflammation and cardiovascular disease processes, including hypertension, vascular calcification, atherosclerosis, myocardial infarction, and obesity. R568, L-type amino acids, and sinalcaser augment the effect of extracellular calcium ([Ca^2+^]_o_) and other cations on CaSR [17]. Negative metamorphic modulators of CaSR include NPS2143 and Calhex231, which have the opposite effects on calcium mimetics [18]. CaSR, unlike other G protein-coupled receptors, remains in the endoplasmic reticulum or Golgi apparatus after the complete translational modification. In addition, it can be translocated to the cell membrane when activated by signals from relevant extracellular agonists [19]. Thus, it appears that the expression of the receptor itself is altered when agonists are applied.

In the present study, we found that as blood pressure increased and CaSR levels decreased, myocardial hypertrophy and fibrosis increased in SHRs, ultimately exacerbating detrimental cardiac remodeling. CaSR exhibited a potential anti-myocardial hypertrophy effect in neonatal rat studies [20]. However, the study’s conclusions do not apply to adult rats. CaSR may act through different mechanisms as the immune function of the body changes with age and exposure to the external environment. In this study, we used SHRs at different ages to dynamically observe the role of R568 and NPS2143 in hypertensive cardiac remodeling, indicating that CaSR plays an important protective role in hypertensive myocardial remodeling. However, EF and FS were unaffected in any of the groups in the echocardiography results. This difference may be because the level of myocardial remodeling is still in the early compensatory phase, the major functions have not yet been compromised, and early intervention and treatment of myocardial remodeling can effectively stop or even reverse its development.

In adult mammals, macrophages are present in all tissues and are characterized by different patterns. During the development of inflammation, two distinct macrophage polarization states (proinflammatory M1 type and anti-inflammatory M2 type) are observed. During the onset and progression of hypertension, immune cells [such as T cells, macrophages and dendritic cells (DCs)] infiltrate the kidney, perivascular fat or heart [21–24]. It was observed that immune monocytes in hypertensive patients have a strong proinflammatory phenotype [25], but the proinflammatory cytokines tumor necrosis factor-α (TNF-α) and IL-1β secreted by M1Mφs can lead to hypotension by triggering diuresis [26]. However, genetic deletion of TNF-α and IL-1β receptors can attenuate blood pressure elevation during the renin angiotensin system (RAS) activation [27]. Therefore, additional research is necessary to determine the role of macrophage polarization types in cardiovascular disease.

In this study, we investigated the polarization status of macrophages in the peritoneal cavity and myocardial tissues of rats at different ages. There were more M1Mφs than M2Mφs in the peritoneal cavity of SHRs at 16 weeks of age; although all types were elevated in the peritoneal cavity of SHRs at 24 weeks of age, M2Mφs were still less abundant than M1Mφs. When CaSR expression decreased in the myocardial tissue of SHRs, the number of M1Mφs increased. This indicates that abdominal and myocardial tissues in primary hypertension contain more proinflammatory Mφs than anti-inflammatory. The asynchrony of macrophage type switching at different sites may be attributable to resident tissue macrophages responding to changes in the tissue environment by recruiting macrophages from other sources to reach inflamed tissues [28]. However, in many chronic fibrotic diseases, macrophages are predominantly of a proinflammatory phenotype due to the unknown nature of the irritants and the fact that they cannot be eliminated [29]. In hypertensive states, the release of humoral factors caused by prolonged exposure to high pressure, abnormal blood flow and activation of the neuroendocrine system stimulates altered macrophage types in the abdominal and myocardial tissues of SHRs, thereby promoting the development of hypertension and myocardial remodeling. Further investigation is required to determine whether the source is tissue-lagged macrophages or mononuclear cells recruited from the blood, spleen, and bone marrow.

In addition, elevated calcium levels in cardiac tissue increase [Ca^2+^]_i_ levels and cardiac activity. Due to a feedback mechanism, CaSR is continuously activated and reactivated in response to changes in calcium levels, which contribute to the normal contractility of muscle cells. CaSR is also present in cells, including monocytes, macrophages and dendritic cells [30, 31]. It was observed that [Ca^2+^]_o_ cause an increase in [Ca^2+^]_i_ level, causing CaSR in macrophages to detect pathogens or tissue damage [32]. This is consistent with our experimental findings that R568 enhanced intracellular calcium fluorescence intensity and CaSR protein expression in RAW264.7 cells while inhibiting macrophage polarization toward M1Mφs. However, the application of NPS2143 had the opposite effect. Overall, CaSR reduces blood pressure and improves myocardial remodeling through the involvement of distinct macrophage phenotypes. Actually, this may be related to excess [Ca^2+^]_i_, has been shown to be involved in the modulation of apoptosis [33, 34]. Moreover, studies have demonstrated that melamine-stimulated CaSR mediated Ca^2+^ signaling resulted in a sustained Ca^2+^ entry, which can prolong the rise in [Ca^2+^]_i_. This mechanism might produce an endoplasmic reticulum stress response, thus resulting in reactive oxygen species generation which can produce a caspase mediated apoptosis pathway leading to tubular cell injury [35].

Activation of NLRP3 inflammasome can be induced by “classical” and “nonclassical” pathways. In the classical pathway, NLRP3 inflammasome activation is followed by upregulation of NLRP3 and IL-1β precursors via nuclear transcription factor signaling, recruitment of apoptosis-associated speck-like protein containing a CARD (ASC) and caspase-1 to form a complex [36], producing caspase-l with the activity that promotes downstream production of additional inflammatory mediators (IL-1β, IL-18) as well as synthesis and secretion of chemokines [37].

Ulrich [38] found NLRP3 inflammasome in peripheral blood mononuclear cells of hypertensive patients. Subsequently, studies by Zhu [39] showed that the level of inflammatory response in hypertensive patients can be exacerbated by the activation of NLRP3 inflammasome in immune cells, ultimately affecting the function of the immune cells themselves, and can accelerate the process of myocardial fibrosis and phenotypic transformation of cardiac fibroblasts. We showed that CaSR expression was upregulated, and NLRP3 inflammasome activation and the release of the effector molecule IL-1β were decreased following the application of a CaSR agonist. In addition, we found that R568 combined with the NLRP3 inhibitor MCC950 lowered blood pressure and improved myocardial remodeling without significantly differing from R568 alone.

Recent studies have shown that inhibiting the activation of NLRP3 inflammasome in macrophages improved angiotensin II (Ang II)-induced myocardial remodeling and myocardial fibrosis, but macrophage polarization during this process was not investigated [9]. In a multisystem study, it was observed that inhibiting the activity of NLRP3 inflammasome effectively reduced the polarization of macrophages to M1Mφs and the systemic multiorgan inflammatory response [40–42]. In contrast, our experiments showed that R568, in combination with MCC950, inhibited the macrophage shift to M1Mφs and promoted the shift to M2Mφs, with the effect being comparable to that of R568 alone. It is hypothesized that R568 can inhibit the macrophage transition to M1Mφs and promote their transition to M2Mφs by reducing the activation of the NLRP3 inflammasome and the release of the effector molecule IL-1β.

**FIGURE 7:**
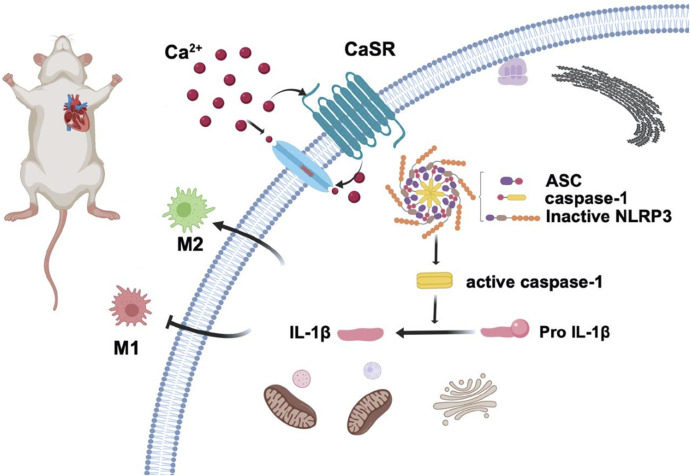
CaSR activation reduces macrophage polarization to the M1-type and increases polarization to the M2-type to reduce blood pressure and improve adverse myocardial remodeling in essential hypertension by reducing NLRP3 inflammasome activation.

Our study shows that CaSR activation can ameliorate adverse myocardial remodeling by inhibiting NLRP3 inflammasome activation and increasing anti-inflammatory macrophages in cardiac tissue (Figure 7). The application of the calcium-mimetic cinacalcet in treating hyperparathyroidism resulted in an improvement in the patient’s cardiovascular system disease in addition to a decrease in blood calcium and phosphorus levels [43]. However, given the complex relationship between CaSR and cardiovascular disease and the fact that most human diseases interact, combining multiple target drugs is likely the most effective treatment. Additional research is still required to develop CaSR-targeting drugs with high specificity.

## Data Availability

The original contributions presented in the study are included in the article/Supplementary Material, further inquiries can be directed to the corresponding authors.
