# Supplementary material for: Calcium-sensing receptor-mediated macrophage polarization improves myocardial remodeling in spontaneously hypertensive rats
Source: Exp Biol Med (Maywood). 2024 Apr 5;249:10112. doi: 10.3389/ebm.2024.10112 (PMC11075494; doi:10.3389/ebm.2024.10112)
Supplement: Supplementary file 1 [file DataSheet1.PDF]

Supplement Figures

Supplement Figure 1

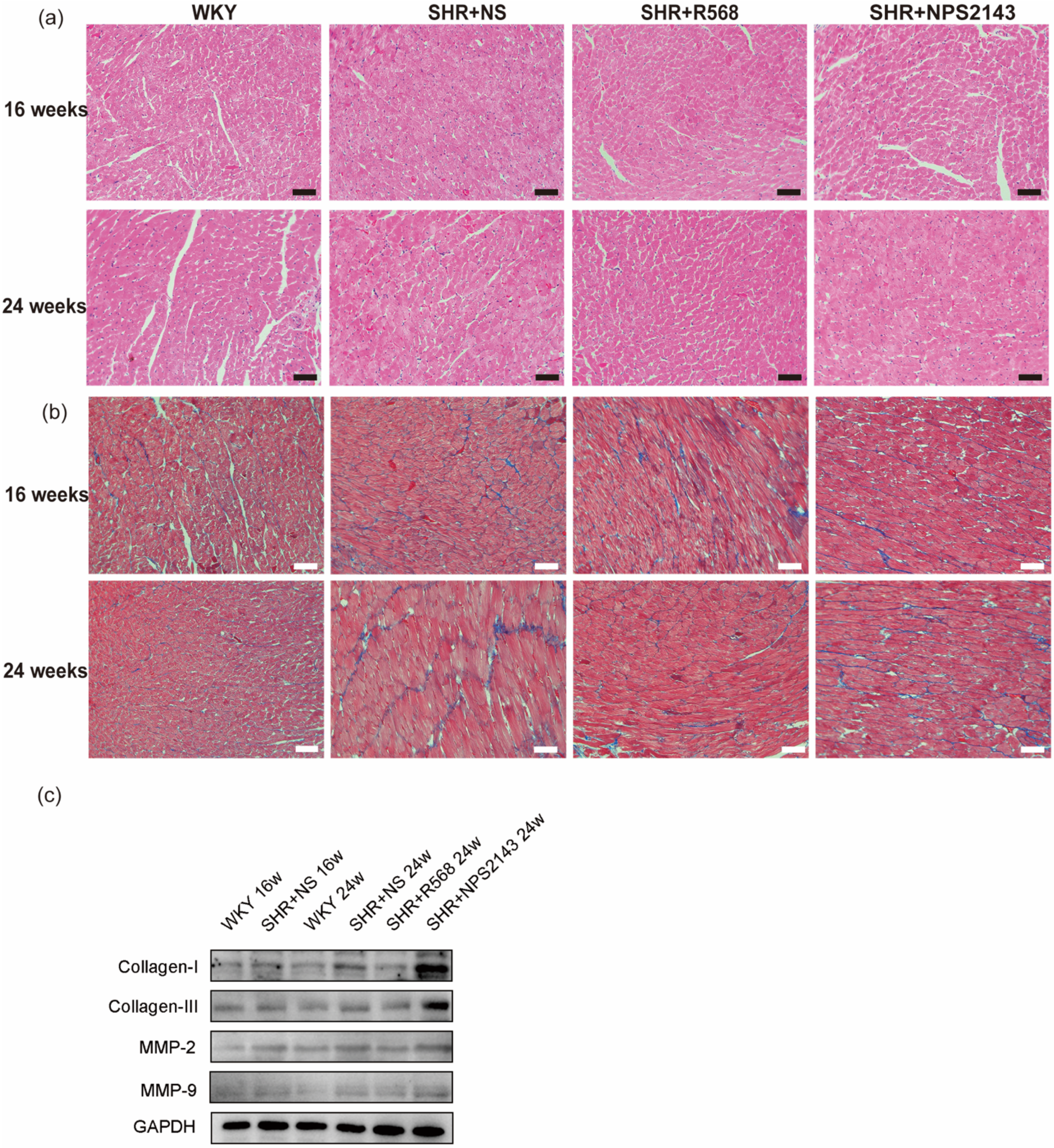

S-Fig 1. (a): representative images of H&E staining; (b): representative images of Masson staining; (c): cardiac-protein expression of left ventricle by western blotting. Images ×100 power, scale bars =100 µm.

Supplement Figure 2

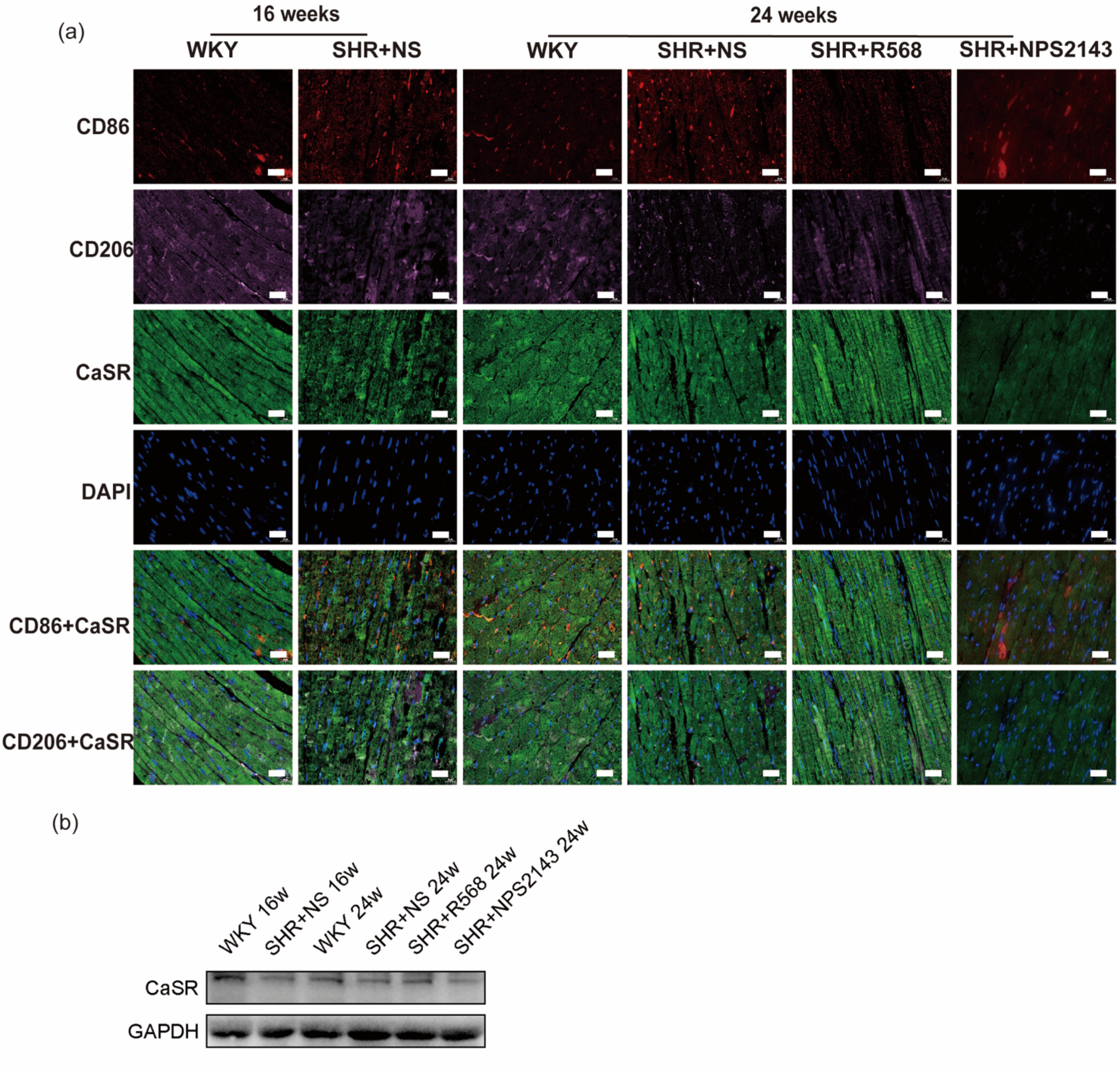

S-Fig 2. (a): immunofluorescence staining (M1Mφ surface marker (CD86, red), M2Mφ surface marker (CD206, pink) and CaSR (green) in the heart with DAPI nuclear staining showing blue color); (b): western blotting of CaSR. Images ×400 power, scale bars =25 μm.

Supplement Figure 3

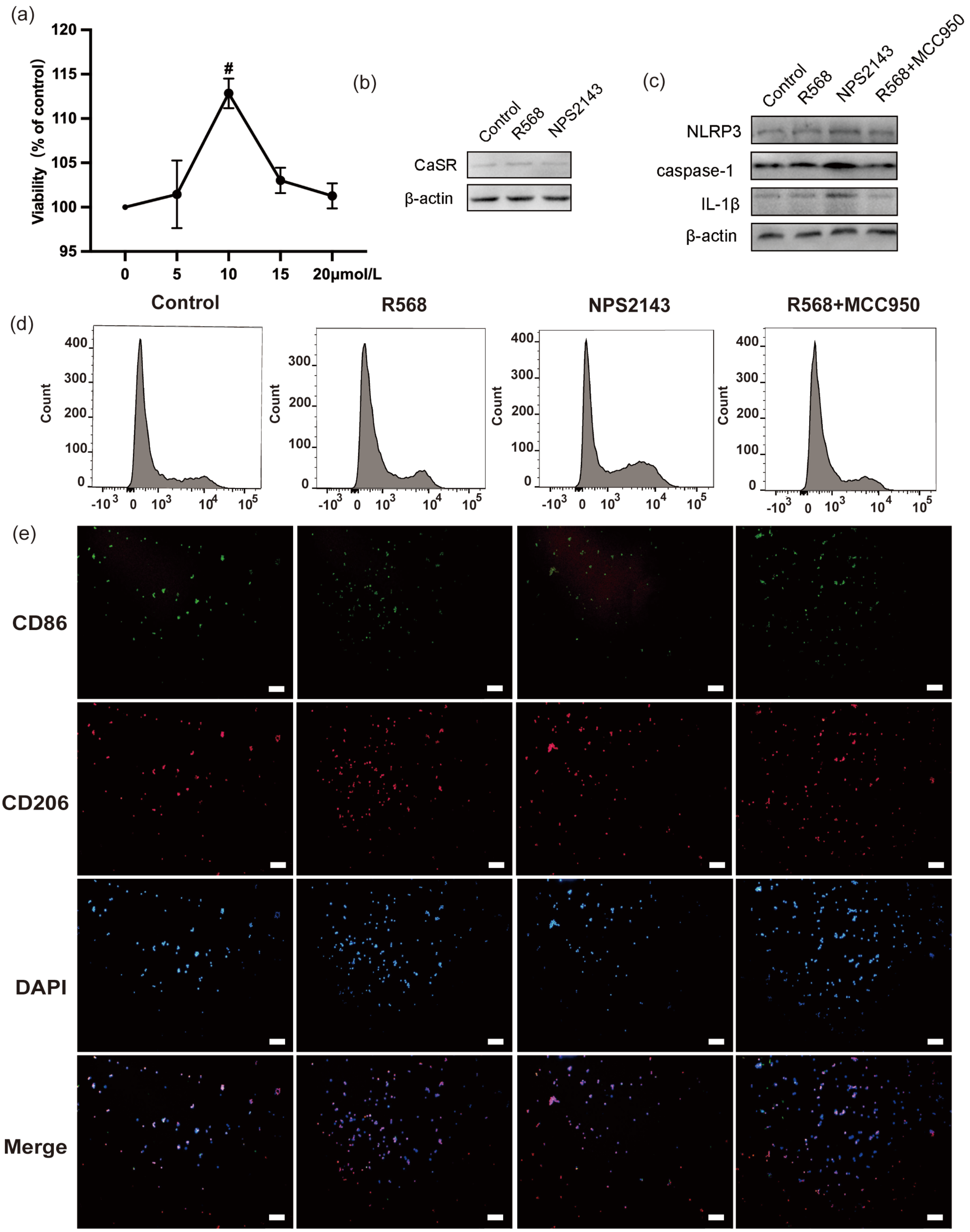

S-Fig 3. (a): Effects of R568 at different concentrations for 2 h on the cell viability ratio of

RAW264.7 cells; (b): western blotting of CaSR; (c): NLRP3 inflammasome-related protein (NLRP3, caspase-1, IL-1β) expression by western blotting; (d): concentration of $[Ca^{2+}]_i$ by flow cytometry; (e): Macrophage types by immunofluorescence staining (M1Mφ surface marker (CD86, green) and M2Mφ surface marker (CD206, red) in the heart with DAPI nuclear staining showing blue color). Images ×100 power, scale bars =100 μm.

Supplement Figure 4

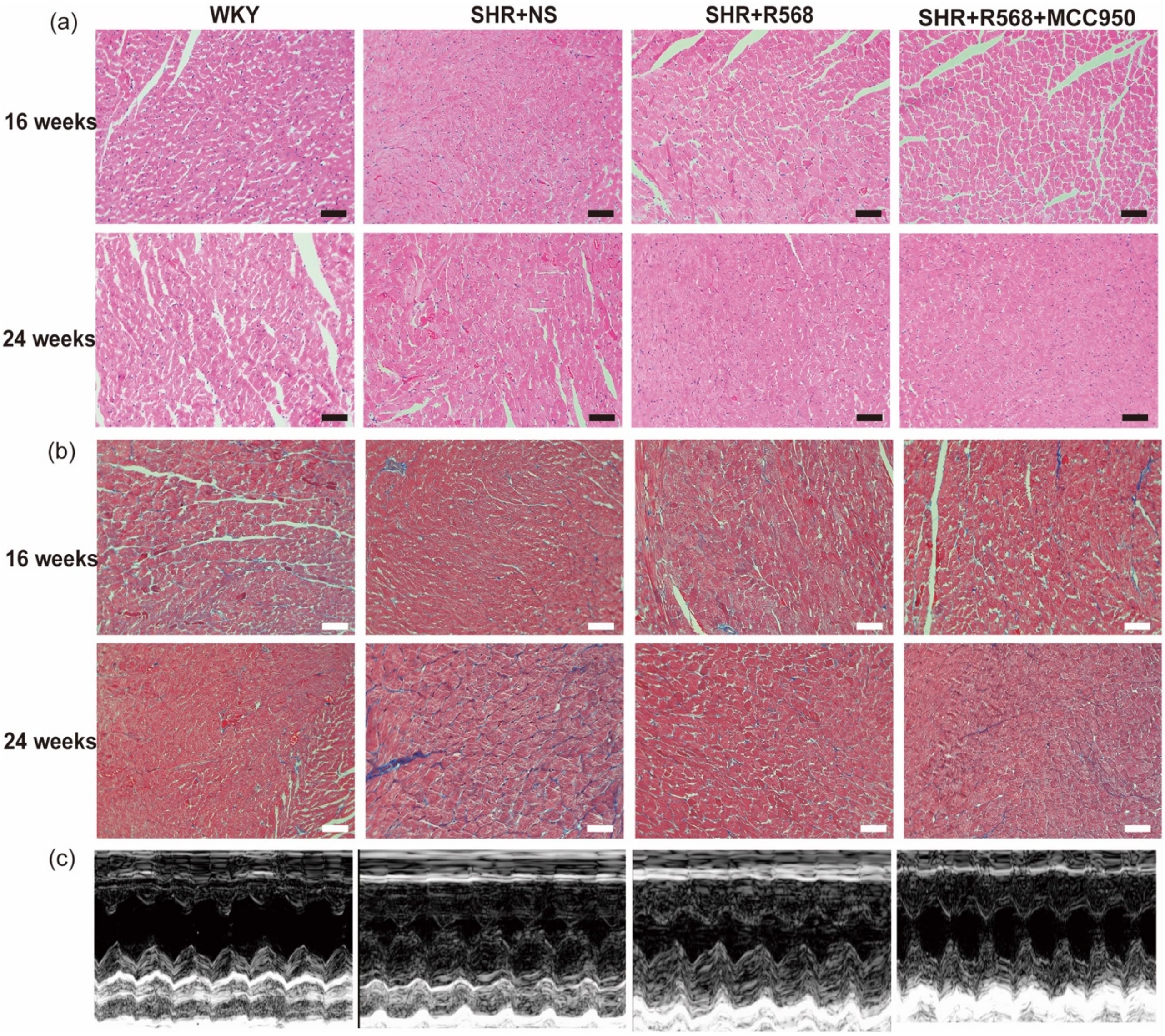

S-Fig 4. (a): representative images of H&E staining; (b): representative images of Masson staining; (c): images of M-mode of LV. Images ×100 power, scale bars =100 μm.

Supplement Figure 5

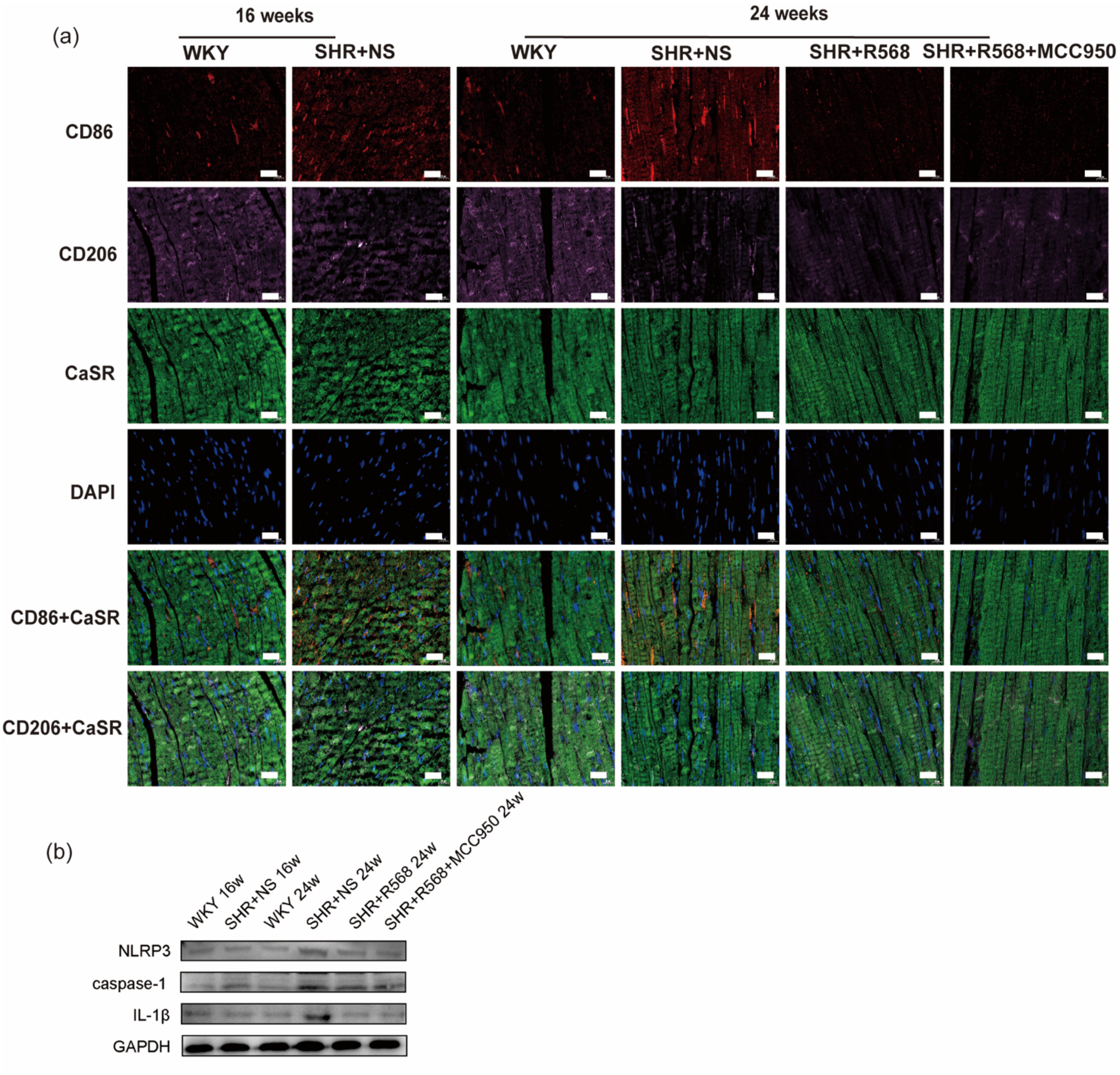

S-Fig 5. (a): macrophage types by immunofluorescence staining (M1Mφ surface marker (CD86, red), M2Mφ surface marker (CD206, pink) and CaSR (green) in the heart with DAPI nuclear staining showing blue color); (b): NLRP3 inflammasome-related protein (NLRP3, caspase-1, IL-1β) expression by western blotting analysis. Images ×400 power, scale bars =25 μm.
